# CTLA4 Message Reflects Pathway Disruption in Monogenic Disorders and Under Therapeutic Blockade

**DOI:** 10.3389/fimmu.2019.00998

**Published:** 2019-05-16

**Authors:** Josselyn E. Garcia-Perez, Ryan M. Baxter, Daniel S. Kong, Richard Tobin, Martin McCarter, John M. Routes, James Verbsky, Michael B. Jordan, Cullen M. Dutmer, Elena W. Y. Hsieh

**Affiliations:** ^1^Department of Immunology and Microbiology, University of Colorado School of Medicine, Aurora, CO, United States; ^2^Division of Surgical Oncology, Department of Surgery, University of Colorado School of Medicine, Aurora, CO, United States; ^3^Division of Asthma, Allergy and Clinical Immunology, Department of Pediatrics, Medical College of Wisconsin, Milwaukee, WI, United States; ^4^Division of Pediatric Rheumatology, Department of Pediatrics, Medical College of Wisconsin, Milwaukee, WI, United States; ^5^Divisions of Immunobiology, and Bone Marrow Transplantation and Immune Deficiency, Cincinnati Children's Hospital Medical Center, University of Cincinnati College of Medicine, Cincinnati, OH, United States; ^6^Division of Allergy and Immunology, Department of Pediatrics, University of Colorado School of Medicine, Children's Hospital Colorado, Aurora, CO, United States

**Keywords:** CTLA4, LRBA, mRNA, ipilimumab, immunotherapy

## Abstract

CTLA-4 is essential for immune tolerance. Heterozygous *CTLA4* mutations cause immune dysregulation evident in defective regulatory T cells with low levels of CTLA-4 expression. Biallelic mutations in *LRBA* also result in immune dysregulation with low levels of CTLA-4 and clinical presentation indistinguishable from CTLA-4 haploinsufficiency. CTLA-4 has become an immunotherapy target whereby its blockade with a monoclonal antibody has resulted in improved survival in advanced melanoma patients, amongst other malignancies. However, this therapeutic manipulation can result in autoimmune/inflammatory complications reminiscent of those seen in genetic defects affecting the CTLA-4 pathway. Despite efforts made to understand and establish disease genotype/phenotype correlations in CTLA-4-haploinsufficiency and LRBA-deficiency, such relationships remain elusive. There is currently no specific immunological marker to assess the degree of CTLA-4 pathway disruption or its relationship with clinical manifestations. Here we compare three different patient groups with disturbances in the CTLA-4 pathway—CTLA-4-haploinsufficiency, LRBA-deficiency, and ipilimumab-treated melanoma patients. Assessment of *CTLA4* mRNA expression in these patient groups demonstrated an inverse correlation between the *CTLA4* message and degree of CTLA-4 pathway disruption. *CTLA4* mRNA levels from melanoma patients under therapeutic CTLA-4 blockade (ipilimumab) were increased compared to patients with either *CTLA4* or *LRBA* mutations that were clinically stable with abatacept treatment. In summary, we show that increased *CTLA4* mRNA levels correlate with the degree of CTLA-4 pathway disruption, suggesting that *CTLA4* mRNA levels may be a quantifiable surrogate for altered CTLA-4 expression.

## Introduction

The regulation of immune responses to self and foreign antigens balances immune tolerance and antimicrobial defense. The stimulatory and inhibitory signals that govern these immune responses can act as accelerator and brake pedals in a car with imbalance between signals engendering reckless abandon vs. inept inaction. Akin to this analogy, gain of function (GOF) mutations affecting stimulatory signaling pathways—*pushing the throttle*—lead to lymphoproliferation and autoimmunity, as seen with *PIK3CD* (encoding the phosphatidylinositide 3-kinase p110δ subunit); while loss of function (LOF) defects that decrease or abolish inhibitory signaling pathways—*releasing the brakes*—result in similar consequences, as observed in CTLA-4 (cytotoxic T lymphocyte antigen-4) haploinsufficiency and LRBA (lipopolysaccharide-responsive and beige-like anchor) deficiency. Although disturbances in stimulatory and inhibitory signals, commonly referred to as immune checkpoints, can naturally occur in the setting of *de novo* or inherited mutations, comparable perturbations can be induced by immunotherapy (IT) used to control autoimmunity and malignancy. Whether the result of a monogenic disorder or a therapeutic intervention, each scenario provides opportunities to investigate mechanisms underlying immune dysregulation that could improve management strategies in primary immunodeficiency, autoimmunity, and malignancy.

CTLA-4 is constitutively expressed in regulatory T cells (Tregs) and can be induced in conventional T cells (Tcon) (1). CTLA-4 competes with CD28 to bind the costimulatory CD80 (B7-1) or CD86 (B7-2) receptors on antigen presenting cells, and upon binding, it stimulates the suppressive function of Tregs (2). Consequently, defects in CTLA-4 protein expression or trafficking pathways result in immune dysregulation (3, 4), as evidenced by CTLA-4 haploinsufficiency in humans and *Ctla4*^−/−^ mice—both of which show decreased frequency of Tregs, multiorgan lymphocytic infiltration, and autoimmunity (4, 5). It is important to note that human biallelic CTLA-4 deficiency has not been described, and that *Ctla4*^+/^^−^ mice do not show signs of overt disease. LOF biallelic mutations in *LRBA* also cause immune dysregulation, with manifestations similar to CTLA-4 haploinsufficiency. In fact, patients with LRBA deficiency often present with low levels of CTLA-4 surface expression, given that the lack of CTLA-4-LRBA interaction results in increased CTLA-4 transport to lysosomes for degradation (3). Interestingly, *Lrba*^−/−^ mice show no obvious sign of disease, despite low Ctla-4 surface expression on Tregs (6). As an immune checkpoint, CTLA-4 has been used as a therapeutic target in both autoimmunity and malignancy. In autoimmunity, the use of a CTLA-4-Ig fusion protein (abatacept, Orencia) suppresses T cell activation by utilizing the capability of CTLA-4 to bind CD80 and CD86 receptors with higher affinity and avidity than CD28 (7). Conversely, in malignancy, inhibition of CTLA-4 function by an IgG_1_ monoclonal antibody (ipilimumab, Yervoy) enhances T-cell activation leading to tumor regression (8). The use of ipilimumab in melanoma patients improved their overall survival by 45.6% after 1 year of therapy (9). However, reminiscent of CTLA-4-haploinsufficiency, use of ipilimumab has been accompanied by an increase in immune-related adverse effects (56.3% of patients), including enteropathies, pruritus, autoimmune hepatitis, thyroiditis, and arthritis (10, 11).

Whether primary (monogenic) or iatrogenic in nature, dysregulation of the CTLA-4 signaling pathway leads to similar autoimmune/inflammatory complications. However, a side-by-side immunological comparison of these patients has not been previously performed. Here, we present an immunological evaluation of CTLA-4 haploinsufficient, LRBA deficient, and ipilimumab-treated melanoma patients, focusing on the degree of CTLA-4 disruption in each patient category.

## Dysregulated CTLA-4 Expression in CTLA-4 haploinsufficiency and LRBA Deficiency

Kuehn et al. first described CTLA-4 haploinsufficiency in seven patients from four unrelated families, where affected patients had reduced CTLA-4 protein and mRNA expression despite normal Treg frequency (12). In general, the patients had autoimmune cytopenia, lymphoproliferation, and lymphocytic infiltration of non-lymphoid organs, leading investigators to call the disorder by CTLA-4-haploinsufficiency with autoimmune infiltration (CHAI) disease. However, there was a wide spectrum of disease manifestations and incomplete penetrance, including asymptomatic mutation carriers (12, 13). Interestingly, both asymptomatic and symptomatic mutation carriers had lower CTLA-4 protein levels than healthy controls, demonstrating that in order to drive healthy CTLA-4 expression, two functional alleles appeared necessary (10). Subsequent reports of other CTLA-4 haploinsufficiency patients have confirmed and expanded the clinical variability seen in these cohorts, further supporting the lack of association between genotype, phenotype, and penetrance (10, 11). Taken together, the description of these heterozygous patients demonstrates that there is no clear correlation between CTLA-4 protein expression and disease severity or penetrance. Absence of a measurable parameter to predict or quantify the pathogenicity of a *CTLA4* variant in an individual poses a considerable challenge in clinical management, including considerations for disease surveillance, treatment thresholds, and ultimate prognosis.

*CTLA4* has four exons: exon 1 encodes the signal peptide, and mutations in this exon abolish CTLA-4 protein expression (10); exon 2 encodes the dimerization and ligand-binding domains, and mutations in this area impede dimerization and interaction with B7 receptors (10, 13); exon 3 encodes for the transmembrane domain, and mutations in this exon impair ligand binding and uptake (10, 13); exon 4 encodes for the cytoplasmic tail (14). A recent study encompassing 133 patients and 54 unrelated families identified a total of 155 exonic variants (13). While every patient presented with immune dysregulation, the disease phenotype was highly variable and did not correlate with CTLA-4 protein expression. In general, 84% of patients presented with hypogammaglobulinemia, followed by 73% with lymphoproliferation, 59% with gastrointestinal problems, and 59% with cytopenia (13). Most patients presented with reduced CD4^+^ and normal CD8^+^ T cells, an increased percentage of CD4^+^ Foxp3^+^ Tregs, decreased absolute B cell and switched memory B cell counts, and a significant expansion of CD21^lo^ B cells (13). In humans, low levels of CD21 are associated with B cell exhaustion as a consequence of chronic antigen exposure (15). Furthermore, a recent study showed that patients who have an expanded CD21^lo^ B cell population after anti-CTLA4 immunotherapy are more likely to develop autoimmune complications compared to patients without such CD21^lo^ B cell expansion (16). However, whether the increase of CD21^lo^ B cells observed in CTLA-4 haploinsufficiency is a direct consequence of the genetic mutation, a result of chronic infection secondary to the immunodeficiency, or a reflection of CTLA-4 pathway disruption, is not clearly understood. Denoting its role in limiting B cells responses and demonstrating the necessity of CTLA-4 in maintaining B cell homeostasis, *Ctla4*^−/−^ mice have spontaneous T-follicular helper cell differentiation, large germinal centers, and increased autoantibody levels (17).

LRBA is required to maintain normal CTLA-4 surface expression via a process of endosomal recycling. Therefore, patients with *LRBA* LOF mutations demonstrate clinical and immunological similarities with CTLA-4 haploinsufficent patients (3). Unlike *CTLA4, LRBA* mutations are biallelic with complete disease penetrance. *LRBA* has 56 coding exons. It harbors a ConA-like lectin domain that is associated with protein trafficking (18), a PH (pleckstrin homology) domain which helps to localize proteins to the cytosol (19), and a BEACH-WD (Beige And Chediak-Higashi—Tryptophan-aspartic (WD) dipeptide) domain that is implicated in the maintenance of intracellular CTLA-4 in T cells. LRBA serves as a scaffold and interacts with the cytoplasmic tail of CTLA-4, preventing it from being degraded in the lysosomes (3). Correspondingly, patients with mutations in *LRBA* have dramatically reduced CTLA-4 levels due to its rapid lysosome degradation (3, 20, 21). Therefore, addition of lysosomal blocking agent prior to the measurement of CTLA-4 protein expression in Tregs may allow for distinction between CTLA-4 haploinsufficiency and LRBA deficiency (20). Additionally, CTLA-4 functional assays—including CTLA-4 cycling and transendocytosis—may also help distinguish CTLA-4 haploinsufficiency from LRBA deficiency (13). However, it should be noted that studies in larger patient cohorts have shown that both CTLA-4 haploinsufficient and LRBA deficient patients can present with normal CTLA-4 expression levels (13, 22). Similarly to CTLA-4 haploinsufficient patients, LRBA deficient patients have a wide range of phenotypic presentations, including inflammatory bowel disease-like enteropathy, autoimmune hemolytic anemia, and immune thrombocytopenic purpura (23, 24). They also often have hypogammaglobulinemia and increased CD21^lo^ B cells (21, 25). The *Lrba*^−/−^ mouse model, however, did not show signs of overt disease, and had normal T and B cell proliferation and survival despite reduced Ctla-4 protein expression (6, 26).

Despite our current knowledge on the mechanisms by which CTLA-4 regulates T cell activation, how different degrees of disturbance in this pathway relate to varied penetrance and heterogeneous clinical manifestations remains elusive. Below, we compare patients with different degrees of CTLA-4 pathway disruption, from haploinsufficiency of the gene (*CTLA4* mutations), to disrupted trafficking of the protein (*LRBA* mutations), to complete blockade of cell surface protein expression (ipilimumab treatment).

## Two Novel *CTLA4* Splice Variants Result in a Truncated Protein With Reduced CTLA-4 Expression on Tregs

**Figure 1:**
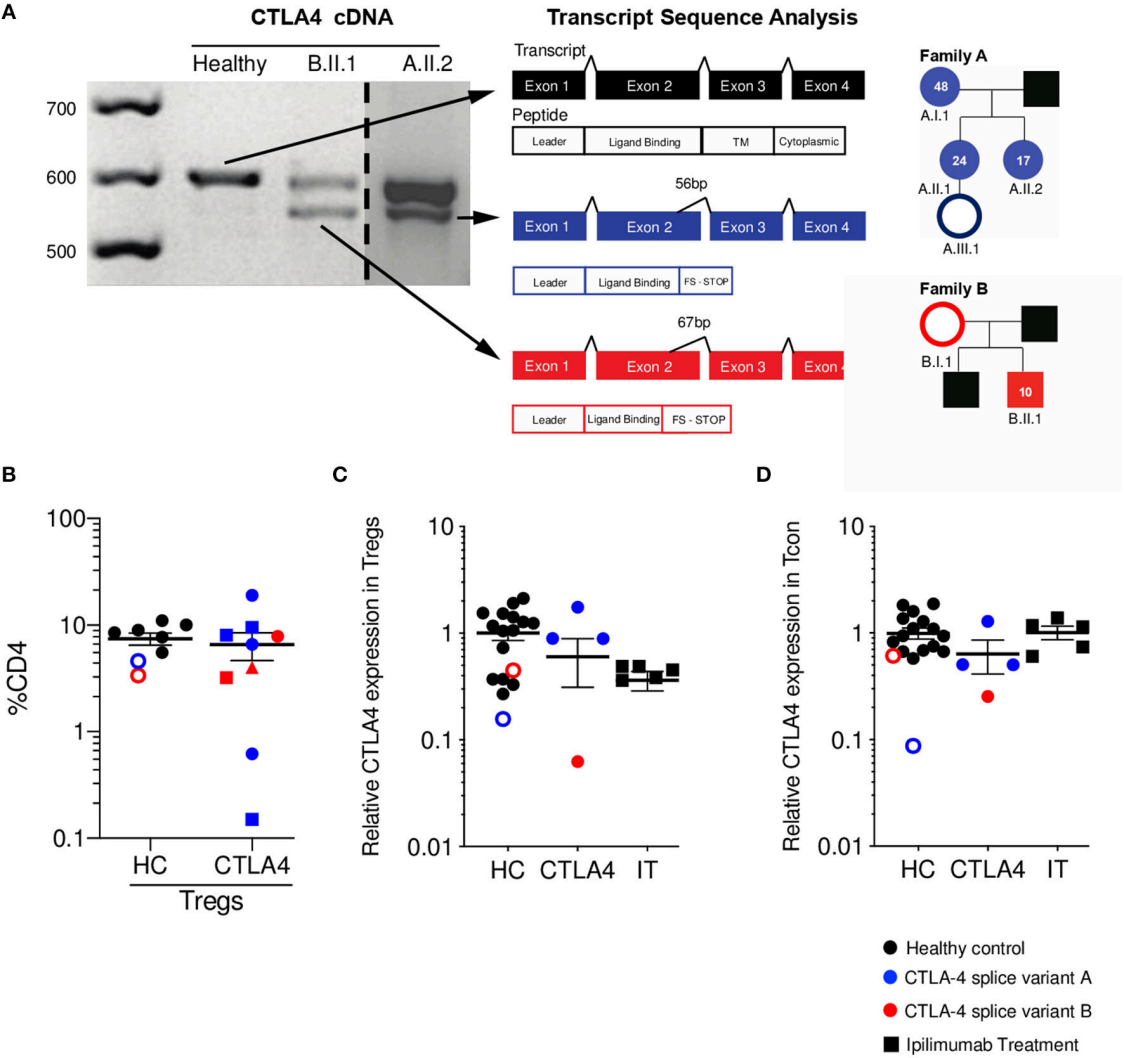
*CTLA4* splice variants result in a truncated protein with reduced expression on Tregs. **(A)** *CTLA4* cDNA amplification from healthy control (HC, black), patient A.II.2 (blue), and B.II.1 (red). Dashed line indicates where a single gel image was spliced to omit irrelevant lanes. Splice variants in patients' alleles are shown in a schematic of the four exons for *CTLA4*. Sanger sequencing of variant bands determined the precise location and size of mRNA deletions. Pedigrees with respective age at diagnosis are shown. Asymptomatic carriers are denoted as unfilled circles. **(B)** Percentage of Tregs (Foxp3+ CD25+ as %CD4+). Different shapes denote different time points. **(C)** Relative CTLA-4 expression on Tregs (patients CTLA-4 MFI normalized to HC CTLA-4 MFI). **(D)** Relative CTLA-4 expression on conventional T cells (Tcon/ CD4+ Foxp3-) (patients CTLA-4 MFI normalized to HC CTLA-4 MFI). (HC *n* = 15 black circles, asymptomatic carriers are red/blue unfilled circles); CTLA-4 *n* = 4 blue is splice variant **A**, Red is splice variant **B**; IT *n* = 5) Average ± SEM is shown.

We identified six individuals from two unrelated families with two novel mutations (family A c.401T>G, Family B c.457G>A) that create cryptic splice sites and introduce frameshifts with premature stop codons in exon 2 (Figure 1A). All members of family A presented with hypogammaglobulinemia, enteropathy, arthritis, and recurrent fungal infections (except A.III.1, who is an asymptomatic carrier at the diagnosis age of 2 months). Patient B.II.1 presented with type I diabetes, autoimmune thyroiditis, and enteropathy; patient B.I.1 is an asymptomatic carrier and was incidentally identified when the proband was sequenced. Immunophenotyping demonstrated similar percentages of Tregs in symptomatic patients compared to healthy controls and asymptomatic carriers (Figure 1B). Additionally, all three adults from family A (A.I.1, A.II.1, A.II.2) had expanded CD21^lo^ B cells per clinical immunophenotyping (data not shown).

As expected, total CTLA-4 levels on Tregs were decreased when compared to healthy controls (Figure 1C). It is important to mention that the asymptomatic family members were included within the HC group, as we define the CTLA-4 group as a combination of those who are genetic mutation-positive and symptomatic. We compared the CTLA-4 protein expression in Tregs (CD4+ Foxp3+) from these patients to five ipilimumab-treated melanoma patients, since they also have disruption of the CTLA-4 pathway and can experience a wide array of autoimmune complications. Of note, two of the ipilimumab-treated patients developed diarrhea, fever, nausea, pruritis, and autoimmune hepatitis. CTLA-4 haploinsufficient patients show a wide range of CTLA-4 expression in Tregs, as shown in previous studies (13, 20), while ipilimumab-treated patients demonstrate markedly decreased CTLA-4 protein expression when compared with healthy controls (Figure 1C). Since CTLA-4 can also be expressed in Tcon (CD4+ Foxp3-), we also measured its levels in this population. While patients with monogenic CTLA-4 pathway defects had generally reduced levels, ipilimumab-treated patients showed levels similar to that of healthy controls (Figure 1D).

## *CTLA4* mRNA Expression Inversely Correlates With the Degree of CTLA-4 Pathway Disruption

As expected from previous literature, our data showed that neither Treg CTLA-4 protein expression levels nor Treg frequency correlated with clinical presentation severity. However, when *CTLA4* mRNA expression levels from peripheral blood mononuclear cells (PBMCs) were evaluated, the relative quantification of mRNA seemed to correlate with degree of CTLA-4 pathway disruption. To make this an unbiased assessment, and considering how LRBA deficiency evokes a CTLA-4 haploinsufficiency phenotype, we included two patients with mutations in *LRBA* (c.4334G>A; p. 1445 R>Q and c. 4333C>T; p. R1445^*^, respectively) that presented with interstitial lung disease, psoriasis, and enteritis. We also included one novel *CTLA4* mutation in exon 2 (c. 380A>G; p. 127 Y>C), one known *CTLA4* mutation in exon 2 (c.208C>T; p.70R>W) (10), one novel deletion in exon 1 (c. 90_104del15; p. I31_C35del), one novel frame shift deletion in exon 2 (c. 255_256del; p. A86_fs), and one novel mutation in the invariant AG acceptor splice site of intron 3 (c. 458-1G>C).

**Figure 2:**
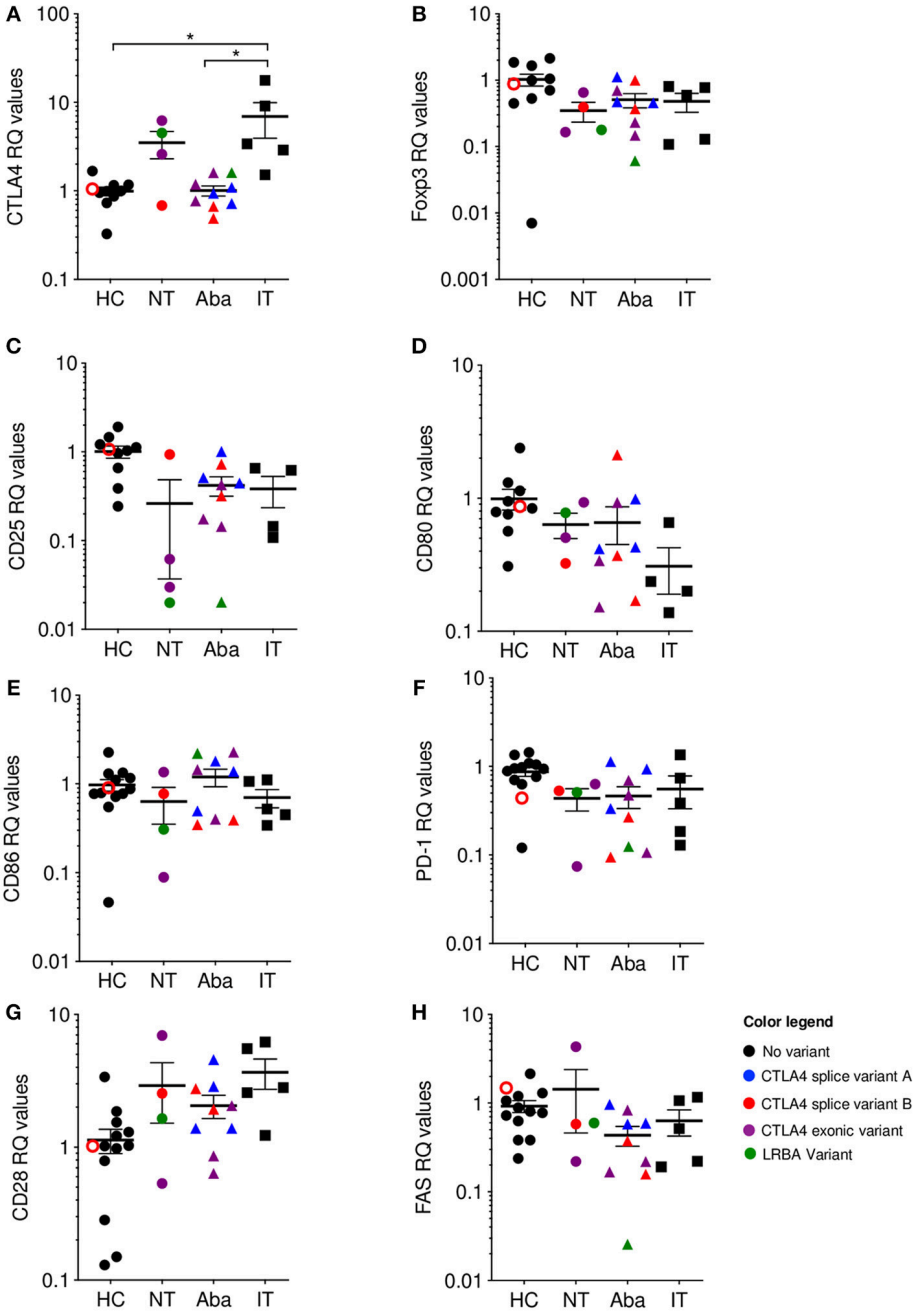
*CTLA4* mRNA levels are correlated with phenotype severity. qPCR Relative quantification (RQ) values where color is associated with genotype: Healthy (HC)/No mutation: black; *CTLA4* splice variant family **(A)** blue; *CTLA4* splice variant family **(B)** red; *CTLA4* exonic variants: purple; *LRBA* variants: green. Unfilled circles are asymptomatic carriers. Shape is associated with treatment; circles: no treatment (NT), triangles: abatacept therapy (Aba), and squares: ipilimumab therapy (IT). Patients in the NT and Aba groups represent different individuals in each group. Exonic variants in NT: 90_104del15 and c. 255_256del the other variants in Aba group. **(A)** CTLA-4, **(B)** Foxp3, **(C)** CD25, **(D)** CD80, **(E)** CD86, **(F)** PD-1, **(G)** CD28, and **(H)** FAS. Average ± SEM is shown. ^*^p < 0.05.

Independent of CTLA-4 haploinsufficiency or LRBA deficiency status, abatacept non-treated (NT) patients showed elevated levels of *CTLA4* mRNA expression, while abatacept-treated (Aba) patients had *CTLA4* mRNA levels closer to healthy control levels (HC). Abatacept-treated patients refer to patients who have received the standard loading dose of 10 mg/kg intravenously on day 1, followed by 125mg subcutaneously weekly, for at least three treatment doses. Ipilimumab-treated (IT) patients showed a significant *CTLA4* mRNA level increase when compared to abatacept-treated patients, suggesting that CTLA-4 pathway disruption leads to mRNA overexpression (Figure 2A). Ipilimumab-treated patients refer to patients who received standard dosing of 10 mg/kg intravenously for at least three treatment cycles. Furthermore, the two most elevated values in the abatacept non-treated patients belong to patients with the most severe disease presentation: one with an exonic *CTLA4* mutation (purple) and the other with an *LRBA* mutation (green). Interestingly, ipilimumab-treated patients (black squares), who have an iatrogenic disruption in CTLA-4 secondary to anti-CTLA-4 monoclonal therapy, had the most elevated *CTLA4* mRNA expression levels of all the patients evaluated (Supplementary Table 1). Additionally, the melanoma patients presenting the most severe immune-related adverse events (diarrhea, nausea, pruritic rash, amongst others) due to ipilimumab treatment, demonstrate the highest *CTLA4* mRNA fold change Supplementary Table 1. To ensure this finding in increased *CTLA4* mRNA expression did not merely reflect T cell activation, we evaluated mRNA expression of several T cell activation markers such as CD25, PD-1, and FAS; and T cell markers involved or related to the CTLA-4 pathway, including CD80, CD86, and CD28. mRNA expression levels for these markers in the abatacept-treated and non-treated patients were comparable to those of healthy controls, suggesting that the correlation between *CTLA4* mRNA expression levels with the degree of CTLA-4 pathway disruption is likely independent of T cell activation status, and may be intrinsic to the dysregulation of this pathway (Figures 2B–H). While *CTLA4* transcription regulation in human lymphocytes is still poorly understood, Gibson et al. identified NFAT as a regulator of the *CTLA4* gene (27). Future studies evaluating *CTLA4* transcription regulation in the patients treated with anti-CTLA-4 immunotherapy could shed light onto this inverse correlation observed between *CTLA4* mRNA levels and the degree of CTLA-4 pathway disruption. CTLA-4 is upregulated following stimulatory signals from TCR:MHC and CD28:B7 interactions (28). We speculate that cells increase *CTLA4* transcription in response to the stimulatory signals from TCR:MHC binding, but failure to establish intact CTLA-4 signaling, due to haploinsufficiency or anti-CTLA4 immunotherapy, results in sustained accumulation of the transcript.

## Closing Remarks

CTLA-4 is an important immune checkpoint that modulates tolerance, in which CTLA-4 pathway engagement promotes Treg suppressive function, while its blockade enhances T cell activation. In the absence of a competently functioning CTLA-4 pathway, autoimmunity ensues. Despite differing in genetic origins, the irrevocably linked biology of CTLA-4 and LRBA leads CTLA-4 haploinsufficiency and LRBA deficiency patients to similar clinical ends. Although there have been many attempts to understand underlying disease mechanisms in CTLA-4 haploinsufficiency and LRBA deficiency and correlate genotype with phenotype, none have been successful thus far (13, 29). Similarly, investigations into predictive parameters or disease biomarkers that might identify those patients at risk of developing autoimmune complications after CTLA-4 blockade have been unrevealing. In this perspective, we observe an apparent correlation between *CTLA4* mRNA levels and the degree of CTLA-4 pathway disruption. In CTLA-4 haploinsufficient and LRBA deficient patients, those that are abatacept non-treated show higher levels of *CTLA4* mRNA expression than abatacept-treated patients, who show *CTLA4* mRNA expression levels similar to healthy controls. Consistent with this correlation of CTLA-4 pathway disruption, ipilimumab-treated patients demonstrate the highest *CTLA4* mRNA expression levels compared to the monogenic patients or healthy controls; particularly, the ipilimumab-treated patients who developed autoimmunity were those who had the highest *CTLA4* mRNA expression (most CTLA-4 pathway disruption). These findings represent the first study comparing genetic vs. therapeutic disruptions to the CTLA-4 pathway and suggest the evaluation of *CTLA4* mRNA expression as a proxy for severity of CTLA-4 pathway disruption in patients receiving anti-CTLA-4 immunotherapy. Future studies evaluating larger patient cohorts are required to further expand this finding and potentially uncover a reliable biomarker for clinical management of these disorders.

## Materials and Methods

### Study Approval

Human samples were obtained from the Allergy and Immunology Clinic at Children's Hospital Colorado. Age appropriate consent and assent was obtained. All human donors were enrolled under study protocol 16-0918, approved by the Institutional Review Board of the Research Compliance Office at University of Colorado. Stage III melanoma patients were recruited at the University of Colorado Cancer Center Cutaneous Oncology Clinic as part of the clinicaltrials.gov registered clinical trial NCT02403778. All patients provided a written informed consent, and the treatment protocol was approved by the Colorado Multiple Institutional Review Board (#14-0948).

### RT-PCR

PBMCs were isolated from whole blood by Ficoll gradient. CD4+ T cells were negatively selected using MojoSort streptavidin magnetic beads (Biolegend, 76447) coupled to biotinylated antibodies against CD8 (RPA-T8), CD123 (6H6), CD11b (ICRF44), and CD19 (HIB19) (Biolegend, 301004, 306004, 301304, 302204, respectively). CD4+ T cells were further stimulated and enriched by Dynabeads (Thermo Fisher, 11161D) for 24 h at 37°C. RNA from CD4+ T cells was isolated by standard Trizol protocol. First-strand cDNA was generated using SuperScript IV (Thermo Fisher, 18091050). The following primers were designed and ordered from Integrated DNA Technologies (Coralville, IA) FWR primer 3′ atggcttgccttggatttcagcgg 5′, and REV primer: 3′tcacattctggctctgttgggggc 5′. Amplification products were ran on 2.5% agarose gel to resolve predicted splice variants.

### Sanger Sequencing

RT-PCR products were gel extracted with a QIAquick kit (Quiagen 28704) and Sanger sequenced (Eton Bioscience, San Diego, CA) with the same primers used for amplification. Splice sites were confirmed using Benchling software (San Francisco, CA).

### Flow Cytometry Analysis

PBMCs were isolated from whole blood by Ficoll and incubated with 1 ug/mL anti-CD3 (clone UCHT1, BD 555329) for 24 h at 37°C to enrich T cells and induce CTLA-4 expression. Enriched T cells were stained with anti-CD4-PB (Biolegend 344620), and anti-CD25-FITC (Biolegend 302604). Anti-CLTA4-APC (Biolegend 349908), or mouse IgG1-APC isotype control (Biolegend 400120) was applied either before (surface) or after (total) permeabilization. Permeabilization and Foxp3 staining was performed with Transcription Factor Staining Buffer Set (eBioscience 00-5523-00). Data were acquired in a BD LSR X20 flow cytometer (BD Biosciences), all data was analyzed with FlowJo software (TreeStar, Ashland, Ore).

### qPCR

RNA was isolated from PAXgene Blood RNA tubes (PreAnalytix, Hombrechtikon, Switzerland) or Trizol, and cDNA was created with GoScript Reverse Transcription System (Promega, Wisconsin, US) according to manufacturer's recommendations. The following probes were designed and ordered from Integrated DNA Technologies (Coralville, IA): CTLA4 Hs.PT.58.19821900; CD28 Hs.PT.56a.25151111.g; CD80 Hs.PT.56a.38577902; CD86 Hs.PT.58.2389518.g; FAS Hs.PT.56a.4466990; PD1 Hs.PT.58.3719940.g; Foxp3 Hs.PT.58.3671186; CD25 Hs.PT.58.2187899; ACTB Hs.PT.39a.22214847.

### Statistical Analysis

Statistical analysis was performed with Prism software (GraphPad Software, La Jolla, Calif). The Shapiro-wilk test was used to determine normal distribution of the samples. Kruskal-Wallis test was used.

## Ethics Statement

Human samples were obtained from the Allergy and Immunology Clinic at Children's Hospital Colorado. Age appropriate consent and assent was obtained. All human donors were enrolled under study protocol 16-0918, approved by the Institutional Review Board of the Research Compliance Office at University of Colorado. Stage III melanoma patients were recruited at the University of Colorado Cancer Center Cutaneous Oncology Clinic as part of the clinicaltrials.gov registered clinical trial NCT02403778. All patients provided a written informed consent, and the treatment protocol was approved by the Colorado Multiple Institutional Review Board (#14-0948).

## Author Contributions

JG-P and RB: methodology and resources. JG-P, RB, and DK: validation, formal analysis, investigation, and writing original draft. RT, MM, JR, JV, MJ, CD, and EH: writing-review and editing. EH and CD: supervision, project administration, and funding acquisition.

### Conflict of Interest Statement

The authors declare that the research was conducted in the absence of any commercial or financial relationships that could be construed as a potential conflict of interest.

## Supplementary Material

Supplementary Table 1 Demographics Ipilimumab treated patients: Table shows all patients treated with ipilimumab disease stage, immune-related adverse events and their CTLA4 relative quantification.
