# Supplementary material for: CTLA4 Message Reflects Pathway Disruption in Monogenic Disorders and Under Therapeutic Blockade
Source: Front Immunol. 2019 May 16;10:998. doi: 10.3389/fimmu.2019.00998 (PMC6532297; doi:10.3389/fimmu.2019.00998)
Supplement: Supplementary Table 1 — Demographics Ipilimumab treated patients: Table shows all patients treated with ipilimumab disease stage, immune-related adverse events and their CTLA4 relative quantification. [file Table_1.pdf]

Age	Sex	Race	Disease Stage	Improved?	Measure	ir AE	Grade (CTCAE)	Relative Quantification CTLA4
49	F	White	III T3b N2a M0	No Progression	PET CT	Rash	2	2.88
						Nausea	1	
33	M	White	III T2a N2a M0	Progression	PET CT	Diarrhea	3	9.11
						Elevated ALT	3	
						Auto Immune Hepatitis	3	
						elevated AST	2	
						Vomiting	1	
						Nausea	1	
						Pruritis	1	
						Fever	1	
						Fatigue	1	
51	F	White	III T4a N2c M0	No Progression	PET CT	Pruritic Rash	1	3.4
						Wheezing	1	
						Headaches	1	
66	M	White	III Tx N1b M0	No Progression	PET CT	Anorexia	1	1.516
						Cramps	1	
						Pruritic Rash	1	
66	M	White	IIIc (full staging not completed)	Progression	PET CT	Diarrhea	3	17.763
						Colitis	2	
						Rash	1	
						Decreased Appetite	1	
						Fatigue	1	
						Fever	1	
						Nausea	1	

Demographics Ipilimumab treated patients Table shows all patients treated with ipilimumab disease stage, immune related adverse events and their CTLA4 realtive quantification

Legend
ir AE: immune-related adverse events
CTCAE: Common Terminology Criteria for Adverse Events

Tregs	HC	CTLA4	IT	Tcon	HC	CTLA4	IT	Normalized tc HC
403	1219	3314	1678		397	346	506	3.07052897
404	997	3299	1342		471	344	782	2.11677282
405	1369	6486	1807		460	877	414	2.97608696
	1373		1422		517		948	2.655706
	5244		1812		759		804	6.90909091
407	4722	232			751	173		6.28761651
	3939				645			6.10697674
	2698				568			4.75
	5712				646			8.84210526
	4344				1097			3.95989061
	3892				940			4.14042553
	5656				1290			4.38449612
	7071				1258			5.62082671
	4603				460			10.0065217
	7861				885			8.88248588
419	1662				420			3.95714286
414	582				60			9.7

CTLA4	IT	normalized to	CTL4	IT	3702	Normalized Tc	CTL4
9.57803468	3.31620553	0.32928147	0.89519179	0.4532685		0.58040936	0.50584795
9.59011628	1.71611253	0.26931388	0.89113992	0.36250675		0.68859649	0.50292398
7.39566705	4.3647343	0.36980011	1.75202593	0.48811453		0.67251462	1.28216374
	1.5	0.37088061	0	0.38411669		0.75584795	
	2.25373134	1.4165316	0	0.48946515		1.10964912	
1.34104046		1.27552674	0.06266883	0		1.09795322	0.25292398
		1.06401945				0.94298246	
		0.72879525				0.83040936	
		1.54294976				0.94444444	
		1.17341977				1.60380117	
		1.05132361				1.37426901	
		1.5278228				1.88596491	
		1.91004862				1.83918129	
		1.24338196				0.67251462	
		2.12344679				1.29385965	
		0.44894652				0.61403509	
		0.15721232				0.0877193	

IT	684
0.73976608	
1.14327485	
0.60526316	
1.38596491	
1.1754386	
